# Survival pattern in male breast cancer: distinct from female breast cancer

**DOI:** 10.3389/fonc.2024.1392592

**Published:** 2024-06-28

**Authors:** Sungchan Gwark, Jisun Kim, Il Yong Chung, Hee Jeong Kim, Beom Seok Ko, Jong Won Lee, Byung Ho Son, Sei Hyun Ahn, Sae Byul Lee

**Affiliations:** ^1^ Department of Surgery, Ewha Womans University College of Medicine, Ewha Womans University Mokdong Hospital, Seoul, Republic of Korea; ^2^ Department of Surgery, University of Ulsan College of Medicine, Asan Medical Center, Seoul, Republic of Korea

**Keywords:** breast cancer, male breast cancer, prognosis, overall survival, survival pattern

## Abstract

**Introduction:**

Male breast cancer (MBC) is a rare condition, and recent research has underscored notable distinctions between MBC and breast cancer in women. This study aimed to assess and contrast the long-term survival outcomes and disease patterns of MBC patients with those of their female counterparts.

**Methods:**

We analyzed data from 113,845 patients diagnosed with breast cancer who had undergone curative surgery from the Korean Breast Cancer Registry (KBCR) between January 1990 and August 2014 in Seoul, Korea. The five-year overall survival was analyzed according to clinicopathological characteristics.

**Results:**

Among 113,845 patients with breast cancer, 473 MBC cases were included. The median duration of follow-up was 72 months. The median age at diagnosis was 60 and 48 years for MBC and female breast cancer, respectively. Most male patients (92.6%) underwent total mastectomy, while 50.4% of female patients underwent breast-conserving surgery. Among MBC, 63.2% received chemotherapy, and 83.9% of hormone receptor-positive male patients received endocrine therapy. In survival analysis, MBC demonstrated distinct 5-year overall survival patterns compared with female breast cancer, according to age at diagnosis. In women with breast cancer, the younger age group (≤40 years) demonstrated worse 5-year overall survival than did the older age group (>40 years) (91.3% vs 92.7%, p <0.05). While in MBC, the younger age group (≤40 years) demonstrated better 5-year overall survival than did the older age group (>40 years) (97.4% vs 86.4%, p <0.05).

**Discussion:**

In conclusion within this extensive cohort, we have revealed unique survival patterns in MBC that diverge from those observed in women with breast cancer. This study enhances our comprehension of MBC prognosis and can potentially shed light on unresolved questions, paving the way for future research in the realm of MBC.

## 1 Introduction

Male breast cancer (MBC) is rare, comprising roughly 1% of all cancers in men and approximately 1% of the total breast cancer cases worldwide (1–6). Less than 0.2% of cancer-related deaths in men can be attributed to MBC (7, 8). Owing to the exceptionally low incidence of MBC, studies, clinical trials, and the development of new treatment approaches have primarily centered around BC in women. While insights from studies of BC in women undoubtedly offer valuable guidance in the MBC diagnosis and treatment, it is crucial to emphasize the significant molecular and clinicopathologic differences between the two. A notable difference is the age at which BC is typically diagnosed in men, with men generally developing the condition at an older age compared with women (9).

Until recently, MBC was considered similar to its post-menopausal female counterpart, primarily characterized by estrogen receptor (ER) positivity. However, advancements in research and clinical trials have highlighted significant disparities between the two. MBC typically presents at an older age as well as exhibits more frequent lymph node metastases and a higher prevalence of hormone-receptor positive tumors compared with female BC (10, 11). Furthermore, the risk factors for MBC differ slightly; unlike BC in women, MBC is more likely to occur in individuals with a BRCA2 instead of a BRCA1 mutation (12). In addition, a low androgen state is a recognized risk factor for MBC (13). This study aimed to compare the clinicopathologic characteristics, survival outcomes, and disease patterns of MBC patients with those of their female counterparts.

## 2 Methods

### 2.1 Korean breast cancer registry

The Korean Breast Cancer Registry (KBCR) is a prospectively maintained, multi-institutional registry of the Korean Breast Cancer Society. Breast surgeons in 102 teaching hospitals nationwide participate in this program. As of 2004, this registry was estimated to include 50% of all newly diagnosed patients with BC in Korea. Essential data include the patient’s identification number, sex, age, surgical method, and cancer stage based on the American Joint Committee on Cancer classification. Patients’ age at diagnosis, family history, menopausal status, and tumor characteristics such as subtype and histological grades are recorded. For follow-up, patients were divided into four categories: no evidence of disease (NED), with recurrence, alive with disease, and dead. The type of first recurrence (locoregional or distant metastasis) and causes of death have been further categorized.

### 2.2 Patients and study design

This study was supported by the grant “Elimination of Cancer Project Fund” from the Asan Cancer Institute of Asan Medical Center, Seoul (Institutional review board approval no. 2017-1341), and by the Korean Breast Cancer Society. In this population-based study, we used data from KBCR. The key data elements comprised the patient’s identification number, sex, age, surgical approach, and cancer stage, categorized according to the American Joint Committee on Cancer classification. Additionally, information regarding the patient’s age at the time of diagnosis, family medical history, menopausal status, and tumor characteristics, including subtype and histological grades, were documented. Patients with an unknown cancer stage, a prior cancer diagnosis, or lacking follow-up data were excluded from the study.

The initial diagnostic and follow-up assessments comprised a range of procedures, including mammography, breast ultrasound imaging, magnetic resonance imaging, chest radiography, blood sampling, and clinical examinations. The expression levels of estrogen (ER) and progesterone receptors (PR) were evaluated using the Allred score (14). The HER2 status was considered negative if the immunohistochemistry score was either 1+ or 2+, and HER2 amplification was confirmed as negative based on the results of fluorescence or silver *in situ* hybridization (15). The clinical and histopathologic staging adhered to the guidelines outlined in the seventh edition of the Cancer Staging Manual by the American Joint Committee on Cancer (16).

### 2.3 Statistical analysis

The characteristics of BC in both the female and male groups were compared using the chi-squared (χ²) test. The overall survival was analyzed using the Kaplan–Meier method, and the log-rank test was performed to compare various subgroups. Multivariate Cox proportional hazard analysis was performed to calculate the hazard ratios (HRs) with corresponding 95% confidence intervals (CIs) for survival. Variables with *p*-value ≤ 0.2 in the univariate analysis were included in multivariate analysis.

All statistical analyses were performed using IBM SPSS Statistics version 26.0 for Windows (IBM Co. in Armonk, NY, USA). P-values < 0.05 were considered statistically significant.

## 3 Results

### 3.1 Baseline characteristics

The data of 113,845 patients diagnosed with BC between January 1990 and August 2014 in Seoul, Korea, were analyzed. Among these cases, a total of 473 were MBC cases. **Table 1** presents an overview of the baseline characteristics of the patients. In the MBC group, the median age at diagnosis was 60 years, while it was 48 years for females with BC. Approximately 93.6% of MBC patients exhibited hormone receptor-positive tumors. Among the 473 MBC patients with ER, PR, and HER2 status data, 16 (3.4%) had triple-negative BR. Most (41.2%) MBC patients were diagnosed at stage II, followed by diagnosis at stage I (40.2%). Moreover, 92.6% underwent total mastectomy, while 7.4% opted for breast-conserving surgery (BCS). In contrast, 50.3% of female patients selected BCS.

**Table 1:** Baseline characteristics of patients.

Variables	Total	Female	Male	*P*
Age				<0.001
≤40	21310 (18.7)	21264 (18.8)	46 (9.7)	
>40	92535 (81.3)	92108 (81.2)	427 (90.3)	
Operation				<0.001
BCS	56441 (50.3)	56406 (50.4)	35 (7.4)	
Total mastectomy	55835 (49.7)	55400 (49.6)	435 (92.6)	
Unknown	1569	1566	3	
T stage				<0.001
Tis	424 (0.3)	422 (0.3)	2 (0.4)	
T1	61816 (54.3)	61548 (54.3)	268 (56.7)	
T2	44884 (39.5)	44707 (39.5)	177 (37.4)	
T3	5433 (4.8)	5421 (4.8)	12 (2.5)	
T4	1217 (1.1)	1203 (1.1)	14 (3.0)	
Unknown	71	2	2	
N stage				0.458
N0	70486 (61.9)	70208 (61.9)	278 (61.9)	
N1	28708 (25.2)	28586 (25.2)	122 (25.2)	
N2	8243 (7.2)	8200 (7.2)	43 (7.2)	
N3	6406 (5.6)	6376 (5.6)	30 (6.3)	
Unknown	2	2	0	
Histologic grade				<0.001
G1/2	56794 (64.3)	56557 (64.2)	237 (74.5)	
G3	31554 (35.7)	31473 (35.8)	81 (25.5)	
Unknown	25497	25432	155	
Nuclear grade				<0.001
G1/2	48002 (61.5)	47818 (61.5)	184 (70.0)	
G3	30047 (38.5)	29968 (38.5)	79 (30.0)	
Unknown	35796	35586	210	
LVI				<0.001
No	56219 (67.5)	56035 (67.5)	184 (63.9)	
Yes	27125 (32.5)	27021 (32.5)	104 (36.1)	
Unknown	30501	30316	185	
ER				<0.001
Negative	33515 (34.2)	33477 (34.3)	38 (10.0)	
Positive	64459 (65.8)	64118 (65.7)	341 (90.0)	
Unknown	15871	15777	94	
PR				<0.001
Negative	40782 (41.8)	40714 (41.8)	68 (18.1)	
Positive	56898 (58.2)	56591 (58.2)	307 (81.9)	
Unknown	16165	16067	98	
HER2 status				<0.001
Negative	70753 (79.1)	70462 (79.0)	291 (87.7)	
Positive	18727 (20.9)	18686 (21.0)	41 (12.3)	
Unknown	24365	24224	141	
Ki67				<0.001
≤20	34869 (66.5)	34723473 (66.4)	146 (78.1)	
20<	17596 (33.5)	17555 (33.6)	41 (21.9)	
Unknown	61380	61094	286	
Hormone therapy				<0.001
No	26511 (30.6)	26455 (30.6)	56 (16.1)	
Yes	60179 (69.4)	59887 (69.4)	292 (83.9)	
Unknown	27155	27030	125	
Chemotherapy				<0.001
No	23680 (25.4)	23548 (25.4)	132 (36.8)	
Yes	69550 (74.6)	69323 (74.6)	227 (63.2)	
Unknown	20615	20501	114	
Radiotherapy				<0.001
No	34258 (34.4)	33997 (34.2)	261 (74.1)	
Yes	65393 (65.6)	65302 (65.8)	91 (25.9)	
Unknown	14194	14073	121	

Regarding treatment, a total of 91 (25.9%) MBC patients received adjuvant radiation. Among those who underwent BCS, 42.9% received adjuvant radiation, while among those who underwent mastectomy, 14.3% received post-mastectomy radiation (data not shown). In the MBC group, 62.9% received chemotherapy, and 83.7% of hormone receptor-positive male patients were treated with endocrine therapy.

**Table 2** shows the result of a comparative analysis between male and female patients with BC classified according to an age threshold of 40 years. In the group aged 40 years and younger, the proportion of female patients with BC who were ER-negative was higher than that in the group over the age of 40 years (38.2% vs. 33.4%, *p <*0.05); however, in MBC patients, no significant difference was observed, regardless of age (16.7% vs 9.5%, *p* = 0.351). Additionally, the histological (41.8% vs 34.4%) and nuclear grade (43.0% vs 37.5%) in female patients with BC aged 40 years and younger was higher; meanwhile, there was no difference in MBC patients in terms of histologic and nuclear grade.

**Table 2:** Baseline characteristics of patients according to age at diagnosis.

Age at diagnosis	Female			Male		
Variables	≤40	>40	*P*	≤40	>40	*P*
Operation			<0.001			0.765
BCS	10402 (49.7)	46004 (50.5)		4 (8.7)	31 (7.3)	
Total mastectomy	10483 (50.3)	44917 (49.5)		42 (91.3)	393 (92.7)	
Unknown	379	1187		0	3	
T stage			<0.001			0.334
Tis	89 (0.4)	333 (0.3)		0 (0)	2 (0.5)	
T1	104466 (49.2)	51102 (55.5)		28 (60.9)	240 (56.2)	
T2	9067 (42.7)	35640 (38.7)		14 (30.4)	163 (38.2)	
T3	1411 (6.6)	4010 (4.4)		3 (6.5)	9 (2.1)	
T4	235 (1.1)	968 (1.1)		1 (2.2)	13 (3.0)	
Unknown	16	55		0	0	
N stage			<0.001			0.646
N0	12321 (57.9)	57887 (62.9)		24 (52.2)	254 (59.5)	
N1	5932 (27.9)	22654 (24.6)		14 (30.4)	108 (25.3)	
N2	1640 (7.7)	6560 (7.1)		4 (8.7)	39 (9.1)	
N3	1371 (6.5)	5005 (5.4)		4 (8.7)	26 (6.1)	
Unknown	0	2		0	0	
Histologic grade			<0.001			0.198
G1/2	9320 (58.2)	47237 (65.6)		20 (80.0)	217 (74.1)	
G3	6681 (41.8)	24792 (34.4)		5 (20.0)	76 (25.9)	
Unknown	5263	20079		21	134	
Nuclear grade			<0.001			0.653
G1/2	8050 (57.0)	39768 (62.5)		17 (77.3)	167 (69.3)	
G3	6064 (43.0)	23904 (37.5)		5 (22.7)	74 (30.7)	
Unknown	7150	28436		24	186	
LVI			<0.001			0.070
No	9020 (61.8)	47015 (68.7)		12 (57.2)	172 (64.5)	
Yes	5583 (38.2)	21438 (31.3)		9 (42.8)	95 (35.5)	
Unknown	6661	23655		25	160	
ER			<0.001			0.351
Negative	6881 (38.2)	26596 (33.4)		6 (16.7)	32 (9.5)	
Positive	11097 (61.7)	53201 (66.5)		30 (83.3)	311 (90.5)	
Unknown	3286	12491		10	84	
PR			<0.001			0.163
Negative	7595 (42.4)	33119 (41.7)		9 (27.3)	59 (17.2)	
Positive	10303 (57.5)	46288 (58.2)		24 (72.7)	283 (82.8)	
Unknown	3366	12701		13	85	
HER2 status			<0.001			0.001
Negative	12813 (80.2)	57649 (78.7)		17 (70.8)	274 (88.9)	
Positive	3165 (19.7)	15521 (21.2)		7 (29.2)	34 (11.1)	
Unknown	5286	18938		22	119	
Ki67			<0.001			0.348
≤20	5301 (59.9)	29422 (67.7)		11 (64.7)	135 (79.5)	
>20	3536 (40)	14019 (32.2)		6 (35.3)	35 (20.5)	
Unknown	12427	48667		29	257	
Hormone therapy			<0.001			0.056
No	56473 (35.5)	20808 (29.5)		2 (7.2)	54 (17)	
Yes	10252 (64.4)	49635 (70.4)		26 (92.8)	266 (83.0)	
Unknown	5365	21665		18	107	
Chemotherapy			<0.001			0.050
No	3007 (17.3)	20541 (27.2)		6 (19.4)	126 (38.7)	
Yes	14343 (82.6)	54980 (72.7)		25 (80.6)	202 (61.3)	
Unknown	3914	16587		15	99	
Radiotherapy			<0.001			0.104
No	5986 (32.4)	28011 (34.6)		19 (65.5)	242 (74.9)	
Yes	12486 (67.5)	52816 (65.3)		10 (35.5)	81 (25.1)	
Unknown	2792	11281		17	104	

### 3.2 Survival analysis

**Figure 1:**
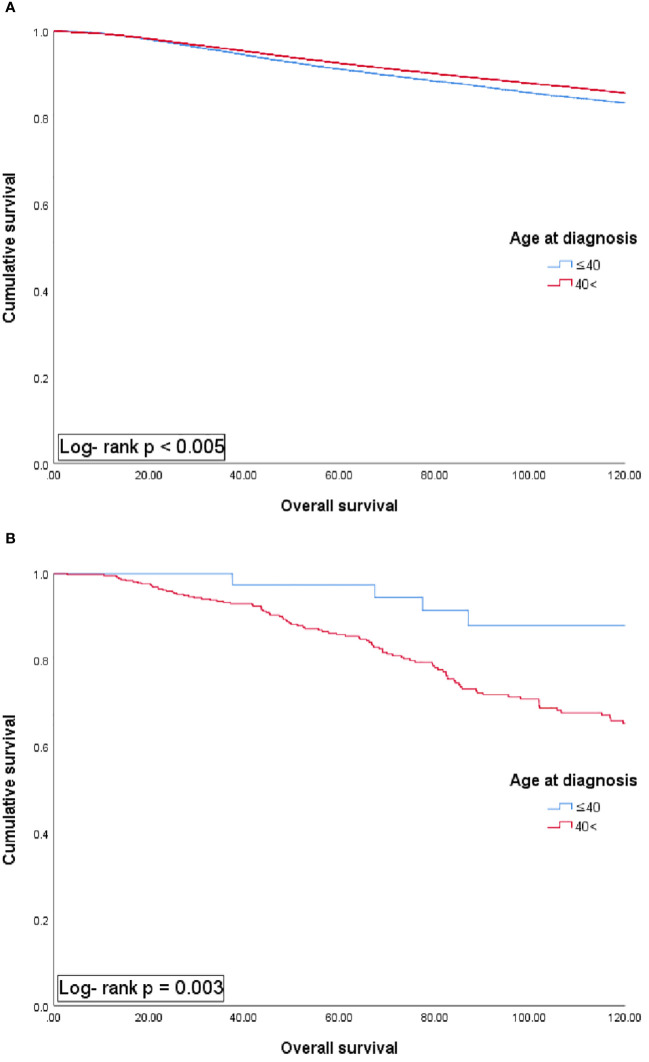
Univariate analysis of overall survival in female **(A)** and male patients with breast cancer **(B)** according to each age group.

The median follow-up duration was 72 months. The 5-year overall survival rate for the entire group was 92.4%. Depending on the age at diagnosis, the MBC patients exhibited distinct patterns in 5-year overall survival compared with that in female patients with BC (**Figure 1**). In female patients with BC, the younger age group (≤40 years) showed a lower 5-year overall survival rate than that in the older age group (>40 years) (91.3% vs 92.7%, *p <*0.05). Conversely, in MBC patients, the younger age group (≤40 years) exhibited a better 5-year overall survival rate compared with the older age group (>40 years) (97.4% vs 86.4%, *p <*0.05).

**Figure 2:**
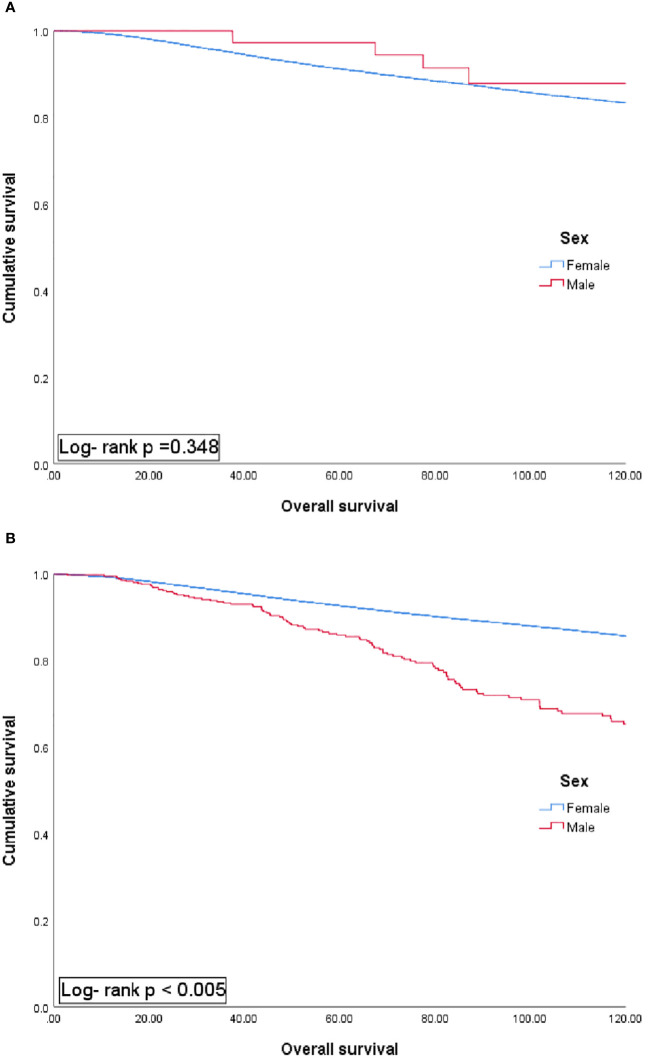
Univariate analysis of overall survival in patients aged 40 years or younger **(A)** and over 40 years **(B)** according to sex group.

The results of the comparative analysis between male and female patients with BC classified based on an age threshold of 40 years showed no significant survival difference between female and male patients in the group under 40 years. Meanwhile, the survival rate of MBC patients was worse in the group aged 40 years and older (**Figure 2**, *p <*0.05).

Factors associated with overall survival in the univariate analysis are presented in **Table 3**. In the male group, the only factor associated with worse overall survival was the age over 40. In the female group, higher TNM stage, ER and PR negativity, and undergoing total mastectomy were associated with worse overall survival, however, the group over the age of 40 years showed better survival (HR 0.94; 95%CI 0.91-0.98; **Table 3**, *p <*0.05).

**Table 3:** Univariate and multivariate cox proportional hazard regression analysis for overall survival.

	Female				Male			
	Univariate analysis		Multivariate analysis		Univariate analysis		Multivariate analysis	
	Hazard ratios (95% CI)	*P*	Hazard ratios (95% CI)	*P*	Hazard ratios (95% CI)	*P*	Hazard ratios (95% CI)	*P*
Age at diagnosis								
≤40	1				1		1	
>40	0.89 (0.85-0.93)	**<0.001**	0.94 (0.91-0.98)	0.007	3.62 (1.47-8.90)	**0.005**	3.46 (1.40-8.54)	0.007
Overall TNM stage								
Stage 1	1				1		1	
Stage 2	2.34 (2.23-2.47)	**<0.001**	2.20 (2.08-2.32)	<0.001	1.21 (0.78-1.88)	0.392	1.20 (0.77-1.88)	0.413
Stage 3	7.15 (6.78-7.54)	**<0.001**	6.55 (6.17-6.95)	<0.001	2.47 (1.56-3.90)	**<0.001**	2.50 (1.57-3.98)	0.000
ER status								
Negative	1				1			
Positive	0.55 (0.53-0.58)	**<0.001**	0.77 (0.73-0.81)	<0.001	1.47 (0.73-2.96)	0.274		
Unknown	0.90 (0.85-0.94)	**<0.001**	0.63 (0.53-0.75)	<0.001	1.72 (0.82-3.58)	0.147		
PR status								
Negative	1				1			
Positive	0.55 (0.53-0.57)	**<0.001**	0.73 (0.70-0.77)	<0.001	0.93 (0.55-1.56)	0.791		
Unknown	0.95 (0.90-0.99)	**0.040**	1.19 (1.00-1.40)	0.053	1.12 (0.64-1.96)	0.680		
HER2 status								
Negative	1				1			
Positive	1.32 (1.25-1.39)	**<0.001**	0.98 (0.93-1.02)	0.381	0.77 (0.38-1.56)	0.476		
Unknown	1.52 (1.46-1.59)	**<0.001**	1.25 (1.19-1.32)	<0.001	1.07 (0.72-1.60)	0.712		
Type of surgery								
BCS	1				1			
TM	2.44 (2.33-2.54)	**<0.001**	1.60 (1.52-1.70)	<0.001	1.73 (0.63-4.70)	0.281		
Unknown	1.35 (1.16-1.58)	**<0.001**	1.27 (1.09-1.48)	0.002	2.83 (0.31-25.37)	0.352		
Radiation therapy								
No	1				1			
Yes	0.81 (0.78-0.85)	**<0.001**	0.97 (0.92-1.02)	0.220	1.01 (0.60-1.68)	0.983		
Unknown	1.38 (1.32-1.45)	**<0.001**	0.99 (0.92-1.06)	0.740	0.91 (0.60-1.39)	0.691		
Chemotherapy								
No	1				1			
Yes	1.61 (1.52-1.70)	**<0.001**	0.73 (0.69-0.77)	<0.001	0.88 (0.56-1.37)	0.581		
Unknown	1.69 (1.58-1.80)	**<0.001**	0.65 (0.60-0.71)	<0.001	0.79 (0.48-1.31)	0.379		
Hormone therapy								
No	1				1		1	
Yes	0.60 (0.58-0.63)	**<0.001**	0.84 (0.80-0.89)	<0.001	0.71 (0.43-1.17)	**0.183**	0.80 (0.48-1.35)	0.419
Unknown	0.92 (0.88-0.96)	**<0.001**	1.09 (1.01-1.17)	0.019	0.61 (0.35-1.06)	0.085	0.8 (0.46-1.42)	0.460

Variables with P-value ≤ 0.2 in the univariate analysis were included in multivariate analysis. Bold values are P-values ≤ 0.2, included in the multivariate analysis.

## 4 Discussion

In this study, we demonstrated that MBC patients exhibited a distinct pattern in 5-year-overall survival compared with female BC patients, classified according to age at diagnosis. In female BC patients, the younger age group (≤40 years) demonstrated worse 5-year overall survival compared with the older age group (>40 years) (91.3% vs 92.7%). Meanwhile, in MBC, the younger age group (≤40 years) demonstrated better 5-year overall survival compared with the older age group (>40 years) (97.4% vs 86.4%). These differences could be attributed to several factors. Men are often initially diagnosed with BC at a more advanced stage compared with women. Approximately 10% of MBCs are *in situ* carcinoma, with the remaining 90% being infiltrating ductal carcinoma (2, 5, 6, 8, 10). MBC tends to exhibit more advanced disease characteristics, including larger tumor size, lymph node involvement, and the presence of distant metastases at the time of diagnosis (5, 6, 8, 17–21). Moreover, MBCs typically express the ER and PR and are most commonly found as unilateral tumors (3, 10, 17, 18, 22–24). A common physical examination finding in MBC is nipple retraction or retroareolar mass detection, which may be the first clinical sign of the disease (6).

In general, women diagnosed with BC at a younger age harbor aggressive clinicopathologic features and have been recognized as a unique biologic entity. Colleonia et al. reported a higher percentage of ER- and PR-negative, vascular or lymphatic invasion, and pathologic grade 3 tumors in young patients compared with older women (25). Additionally, young age is an independent predictor of adverse outcomes (26–30). A retrospective study of more than 1,200 women diagnosed with early-stage BC evaluated the relationship between age, typical prognostic factors, treatment, and patient outcome. In multivariate analyses, younger age is a potent independent prognostic factor, including all potential patient, treatment, and pathology variables (28).

In contrast, MBC exhibits a comparatively mild nature, characterized by low-grade features and hormone receptor-positive expression. The age-specific incidence rate curve for MBC consistently increases with advancing age. The age-specific rates for BC in men demonstrate a parallel increase over time, aligning with a pattern indicative of hormone-independent epithelial carcinogenesis (31). Furthermore, certain high-risk conditions, such as Klinefelter’s syndrome, gynecomastia, obesity, and testicular or liver dysfunction (32–35), have been implicated in some MBCs due to excessive hormonal exposures. However, these conditions may only contribute to a small fraction of MBC cases. The mean ages at diagnosis for Klinefelter’s syndrome and gynecomastia are younger than the mean and/or median ages at diagnosis for MBC (32, 35).

More than two-thirds (90%) of MBC patients opted for a mastectomy, which aligns with findings from the previous studies on MBC (10, 36). Conversely, approximately two-thirds of female patients with BC opted for BCS, and one-third underwent mastectomy (37, 38). The difference in treatment options between men and women can be attributed to concerns in men that complete removal of all at-risk breast tissue with sufficient margins may be challenging because of smaller breast size. Additionally, sex-specific differences in cosmetic preferences might play a role. Moreover, MBCs are frequently located centrally and involve the nipple, which often necessitates the removal of the nipple-areolar complex, limiting the potential aesthetic benefits of BCS (39). Nevertheless, the approach to MBC treatment may have followed a pattern similar to BC in women, with more patients opting for mastectomy and potentially avoiding radiation despite the availability of BCS (37, 40).

The importance of using adjuvant endocrine therapy for hormone receptor-positive MBC is underscored by its association with improved overall survival, aligning with prior studies on men (41, 42). While there was an overall increase in the utilization of adjuvant endocrine therapy over the study period, nearly a third of men with ER+ breast cancer did not receive any endocrine therapy. This study could not assess long-term compliance or the duration of endocrine therapy use, both of which may pose an issue in men (43, 44). Further research is warranted to investigate the factors influencing the utilization of adjuvant endocrine therapy, which is the most effective form of systemic therapy for hormone receptor-positive breast cancer.

This study demonstrated that a positive ER status is a worse prognostic factor, which could be attributed to the limited number of ER-negative patients in this cohort. Additionally, the ER-negative cohort may have included patients with low ER expression due to changes in classification over time (45). The previously observed patterns in population-based incidence suggest a significant causal connection between early-onset hormonal events and ER-negative tumors in pre-menopausal women. Conversely, the importance of accumulated lifetime exposures appears to be more pronounced in the context of ER-positive tumors, post-menopausal women with breast cancer, and overall MBC (11).

Our study had certain limitations owing to its retrospective database reliance. When conducting this research based on the KBCS dataset, several limitations become evident, including the non-population-based nature of the dataset, leading to geographical and sociodemographic disparities in case coverage and lack of recurrence data. Similar datasets have reported issues related to the under-ascertainment of treatment-related variables. We did not consider the impact of changes in treatment practices over time when assessing factors associated with overall survival. Furthermore, the relatively small number of patients in certain sub-groups, such as those with hormone receptor-negative status, restricts the generalizability of our findings to the entire MBC population.

## 5 Conclusion

Although MBC is infrequent and frequently overlooked, there is an increasing recognition of the biological distinctions of BC between men and women. These disparities suggest that MBC should be regarded as a unique condition, separate from female breast cancer. Within this cohort, we have demonstrated distinct survival trends in MBC based on age groups, diverging from patterns observed in female BC. Our study advances our understanding of MBC prognosis and can potentially uncover unresolved issues that could guide future research on MBC.

## Data availability statement

The raw data supporting the conclusions of this article will be made available by the authors, without undue reservation.

## Ethics statement

The studies involving humans were approved by University of Ulsan College of Medicine, Asan Medical Center, Seoul, South Korea. The studies were conducted in accordance with the local legislation and institutional requirements. The participants provided their written informed consent to participate in this study.

## Author contributions

SG: Data curation, Formal analysis, Investigation, Methodology, Software, Validation, Visualization, Writing – original draft, Writing – review & editing. JK: Resources, Writing – review & editing. IYC: Conceptualization, Resources, Supervision, Writing – review & editing. HJK: Conceptualization, Resources, Supervision, Writing – review & editing. BSK: Conceptualization, Resources, Supervision, Writing – review & editing. JWL: Conceptualization, Resources, Supervision, Writing – review & editing. BHS: Conceptualization, Resources, Supervision, Writing – review & editing. SHA: Conceptualization, Resources, Supervision, Writing – review & editing. SBL: Conceptualization, Data curation, Funding acquisition, Methodology, Project administration, Resources, Supervision, Validation, Writing – review & editing.
